# {(1*R*,2*R*)-*N*,*N*′-Bis[2-(*N*-methyl­anilino)benzyl­idene]cyclo­hexane-1,2-diamine-κ^2^ *N*,*N*′}dichloridoiron(II)

**DOI:** 10.1107/S1600536811053773

**Published:** 2011-12-17

**Authors:** Yanyu Zhang, Qiaolin Wu, Ying Mu

**Affiliations:** a State Key Laboratory of Supramolecular Structure and Materials, School of Chemistry, Jilin University, Changchun 130012, People’s Republic of China

## Abstract

In the title compound, [FeCl_2_(C_34_H_36_N_4_)], the Fe^II^ ion is coordinated by two Cl atoms and by two N atoms from a (1*R*,2*R*)-*N*,*N*′-bis[2-(*N*-methyl­anilino)benzyl­idene]cyclo­hexane-1,2-diamine ligand in a distorted tetra­hedral geometry. The mol­ecule has approximate *C*_2_ point symmetry. The dihedral angles between the phenyl and benzene rings on either side of the ligand are 64.56 (14) and 65.61 (13)°.

## Related literature

For background to chiral diimine-based catalysts, see: Li *et al.* (1993 ▶). For the application of iron complexes in enanti­o­selective oxidation, see: Muthupandi *et al.* (2009 ▶). For related structures, see: Yan *et al.* (2009 ▶); Chaggar *et al.* (2003 ▶); Sui-Seng *et al.* (2008 ▶, 2009 ▶).
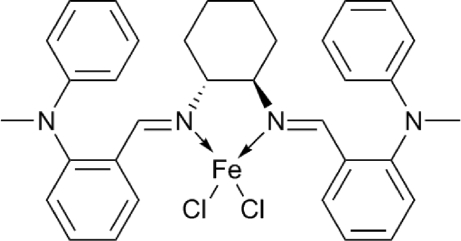


## Experimental

### 

#### Crystal data

[FeCl_2_(C_34_H_36_N_4_)]
*M* *_r_* = 627.42
Orthorhombic, 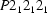

*a* = 13.040 (3) Å
*b* = 13.228 (3) Å
*c* = 20.602 (4) Å
*V* = 3553.6 (12) Å^3^
*Z* = 4
Mo *K*α radiation
μ = 0.60 mm^−1^
*T* = 298 K
0.24 × 0.21 × 0.18 mm

#### Data collection

Rigaku R-AXIS RAPID diffractometer
Absorption correction: multi-scan (*ABSCOR*; Rigaku, 1995 ▶) *T*_min_ = 0.866, *T*_max_ = 0.897
33683 measured reflections
8057 independent reflections
6495 reflections with *I* > 2σ(*I*)
*R*_int_ = 0.043

#### Refinement

*R*[*F* ^2^ > 2σ(*F* ^2^)] = 0.037
*wR*(*F* ^2^) = 0.093
*S* = 1.03
8057 reflections
370 parameters
1 restraint
H-atom parameters constrained
Δρ_max_ = 0.18 e Å^−3^
Δρ_min_ = −0.18 e Å^−3^
Absolute structure: Flack (1983 ▶), 3578 Friedel pairs
Flack parameter: 0.010 (12)

### 

Data collection: *RAPID-AUTO* (Rigaku, 1998 ▶); cell refinement: *RAPID-AUTO*; data reduction: *RAPID-AUTO*; program(s) used to solve structure: *SHELXS97* (Sheldrick, 2008 ▶); program(s) used to refine structure: *SHELXL97* (Sheldrick, 2008 ▶); molecular graphics: *SHELXTL* (Sheldrick, 2008 ▶); software used to prepare material for publication: *SHELXTL*.

## Supplementary Material

Crystal structure: contains datablock(s) I. DOI: 10.1107/S1600536811053773/lh5395sup1.cif

Structure factors: contains datablock(s) I. DOI: 10.1107/S1600536811053773/lh5395Isup2.hkl

Additional supplementary materials:  crystallographic information; 3D view; checkCIF report

## Figures and Tables

**Fig. 1.**
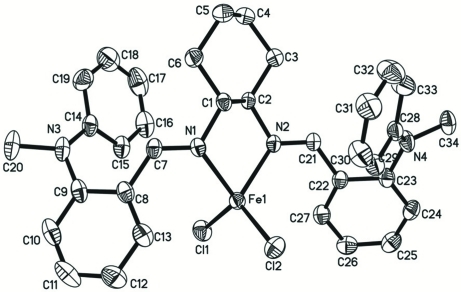
View of the molecule of (I) showing displacement ellipsoids are drawn at the 30% probability level. The hydrogen atoms are omitted for clarity.

## supplementary crystallographic information

### Comment

Asymmetric alkene aziridination with readily available chiral diimine-based catalysts has been studied (Li, *et al.*, 1993). Salen iron complexes were successfully applied in enantioselective oxidation of racemic benzoins (Muthupandi, *et al.* 2009), and iron(II) complexes with tetradentate *PNNP* ligands have been  used as catalysts in the asymmetric hydrogenation of acetophenone (Sui-Seng,*et al.* 2008, 2009). Herein we report a novel chiral iron(II) complex and its molecular structure is shown in Fig. 1.

The title complex (I) possesses approximate *C*_2_ point symmetry with the Fe^II^ ion coordinated in a distorted tetrahedral geometry by two Cl atoms and by two N atoms from the imine groups of the (1*R*,2*R*)-*N*, *N*'-bis[*ortho*- (*N*-methylphenylamino)-benzylidene]-1,2- diaminocyclohexane ligand. The dihedral angles between the phenyl and benzene rings on either side of the ligand are 64.56 (14)° (C8-C13/C14-C19) and 65.61 (13)° (C22-C27C28-C33). The geometric parameters of (I) can be compared to related complexes (Bao *et al.* (2009); Chaggar *et al.* (2003).

### Experimental

[(1*R*,2*R*)-*N*,*N*'-Bis(2-fluorobenzylidene) cyclohexane-1,2-diamine] {L} was prepared according to reported procedure (Li *et al.*, 1993). (1*R*,2*R*)-*N*,*N*'-Bis[*ortho*-(*N*-methylphenylamino)- benzylidene]- 1,2-diaminocyclohexane was synthesized according to the following method: A solution of *n*BuLi (2 mol/*L* in hexane, 30.0 ml, 60.0 mmol) was added to a solution of *N*-methylaniline (6.50 ml, 60.0 mmol) in THF (60 ml) at 195K. The mixture was allowed to warm to room temperature and stirred for 6 h. The resulting solution was transferred into a solution of {L} (9.79 g, 30.0 mmol) in THF (60 ml) at 293K. After stirring for 48 h, the reaction was quenched with H_2_O (20 ml). The organic phase was evaporated to dryness *in vacuo* to give the crude product as a yellow solid. Pure product was obtained by recrystallization from THF as  yellow crystals (11.2 g, 75%) Anal. Calcd for C_34_H_36_N_4_ (500.29): C 81.56, H 7.25, N 11.19; Found: C 81.46, H 7.29, N 11.22%.

The title compound was synthesized according to the following method: FeCl_2_ (127 mg, 1 mmol) was added to a stirred MeCN solution of the ligand (500 mg, 1 mmol) at room temperature. The resulting mixture was stirred for 12 h. The product precipitated as an brown powder and was isolated by filtration (395 mg, 63%). Crystals suitable for X-ray diffraction studies were obtained from a MeCN/Et_2_O solution. Anal. Calcd for C_34_H_36_Cl_2_N_4_Fe (627.43): C 65.09, H 5.78, N 8.93; Found: C 65.14, H 5.69, N 8.90%.

### Refinement

The C-bound H atoms were positioned geometrically with C—H = 0.93 (aromatic and imine carbon), 0.97 (methylene) and 0.96 (methyl) Å, and allowed to ride on their parent atoms in the riding model approximation with *U*_iso_(H) = 1.2 (1.5 for methyl) *U*_eq_(C).

### Crystal data

**Table:**

C_34_H_36_Cl_2_FeN_4_	*D*_x_ = 1.173 Mg m^−^^3^
*M**_r_* = 627.42	Melting point: not measured K
Orthorhombic, *P*2_1_2_1_2_1_	Mo *K*α radiation, λ = 0.71073 Å
Hall symbol: P 2ac 2ab	Cell parameters from 26371 reflections
*a* = 13.040 (3) Å	θ = 3.1–27.5°
*b* = 13.228 (3) Å	µ = 0.60 mm^−^^1^
*c* = 20.602 (4) Å	*T* = 298 K
*V* = 3553.6 (12) Å^3^	Block, brown
*Z* = 4	0.24 × 0.21 × 0.18 mm
*F*(000) = 1312	

### Data collection

**Table:**

Rigaku RAXIS-RAPID diffractometer	8057 independent reflections
Radiation source: fine-focus sealed tube	6495 reflections with *I* > 2σ(*I*)
graphite	*R*_int_ = 0.043
Detector resolution: 0 pixels mm^-1^	θ_max_ = 27.5°, θ_min_ = 3.1°
ω scans	*h* = −16→14
Absorption correction: multi-scan *ABSCOR*, Rigaku (1995).	*k* = −17→16
*T*_min_ = 0.866, *T*_max_ = 0.897	*l* = −26→26
33683 measured reflections	

### Refinement

**Table:**

Refinement on *F*^2^	Secondary atom site location: difference Fourier map
Least-squares matrix: full	Hydrogen site location: inferred from neighbouring sites
*R*[*F*^2^ > 2σ(*F*^2^)] = 0.037	H-atom parameters constrained
*wR*(*F*^2^) = 0.093	*w* = 1/[σ^2^(*F*_o_^2^) + (0.0508*P*)^2^] where *P* = (*F*_o_^2^ + 2*F*_c_^2^)/3
*S* = 1.03	(Δ/σ)_max_ = 0.001
8057 reflections	Δρ_max_ = 0.18 e Å^−^^3^
370 parameters	Δρ_min_ = −0.18 e Å^−^^3^
1 restraint	Absolute structure: Flack (1983) 3578 Friedel pairs
Primary atom site location: structure-invariant direct methods	Flack parameter: 0.010 (12)

### Special details

**Table:**

Experimental. ^1^H NMR (300 MHz, CDCl_3_, 298 K) δ (p.p.m.): 8.27 (s, 2H, C*H*=N), 7.89 (d, 2H, J = 9.0 Hz, Ar*H*), 7.37 (t, 2H, J = 9.0 Hz, Ar*H*), 7.21–7.06 (m, 10H, Ar*H*), 6.74 (t, 2H, J = 9.0 Hz), 6.56 (d, J = 9.0 Hz, 2H), 3.27 (m, 2H, NC*H*CH_2_), 1.03 (s, 6H, NC*H*_3_), 1.75–1.37 (m, 8H,*CH*_2_).
Geometry. All e.s.d.'s (except the e.s.d. in the dihedral angle between two l.s. planes) are estimated using the full covariance matrix. The cell e.s.d.'s are taken into account individually in the estimation of e.s.d.'s in distances, angles and torsion angles; correlations between e.s.d.'s in cell parameters are only used when they are defined by crystal symmetry. An approximate (isotropic) treatment of cell e.s.d.'s is used for estimating e.s.d.'s involving l.s. planes.
Refinement. Refinement of *F*^2^ against ALL reflections. The weighted *R*-factor *wR* and goodness of fit *S* are based on *F*^2^, conventional *R*-factors *R* are based on *F*, with *F* set to zero for negative *F*^2^. The threshold expression of *F*^2^ > σ(*F*^2^) is used only for calculating *R*-factors(gt) *etc*. and is not relevant to the choice of reflections for refinement. *R*-factors based on *F*^2^ are statistically about twice as large as those based on *F*, and *R*- factors based on ALL data will be even larger.

### Fractional atomic coordinates and isotropic or equivalent isotropic displacement parameters (Å^2^)

**Table:**

	*x*	*y*	*z*	*U*_iso_*/*U*_eq_	
Fe1	0.56631 (2)	0.42985 (2)	0.460486 (15)	0.04245 (9)	
Cl1	0.67993 (5)	0.48063 (5)	0.53572 (3)	0.06293 (17)	
Cl2	0.46852 (6)	0.54282 (5)	0.40817 (3)	0.06693 (18)	
N1	0.47736 (13)	0.30362 (14)	0.49010 (8)	0.0402 (4)	
N2	0.63149 (13)	0.31650 (13)	0.40093 (8)	0.0394 (4)	
N3	0.45490 (16)	0.25188 (18)	0.67965 (10)	0.0584 (5)	
N4	0.67088 (15)	0.36560 (17)	0.21363 (10)	0.0566 (5)	
C1	0.48832 (16)	0.21737 (16)	0.44434 (10)	0.0394 (5)	
H1	0.4472	0.2329	0.4058	0.090*	
C2	0.60131 (15)	0.21292 (15)	0.42297 (10)	0.0374 (4)	
H2	0.6419	0.1980	0.4619	0.090*	
C3	0.62008 (19)	0.12705 (19)	0.37537 (12)	0.0506 (6)	
H3A	0.6927	0.1233	0.3653	0.090*	
H3B	0.5833	0.1404	0.3353	0.090*	
C4	0.5846 (2)	0.02654 (18)	0.40358 (13)	0.0597 (6)	
H4A	0.5955	−0.0267	0.3720	0.090*	
H4B	0.6248	0.0108	0.4419	0.090*	
C5	0.4727 (2)	0.0309 (2)	0.42142 (13)	0.0638 (7)	
H5A	0.4519	−0.0333	0.4400	0.090*	
H5B	0.4321	0.0424	0.3827	0.090*	
C6	0.45287 (17)	0.11521 (17)	0.47001 (12)	0.0518 (5)	
H6A	0.4887	0.1003	0.5101	0.090*	
H6B	0.3801	0.1183	0.4795	0.090*	
C7	0.43205 (15)	0.29153 (16)	0.54426 (10)	0.0430 (4)	
H7	0.4067	0.2276	0.5540	0.090*	
C8	0.41725 (16)	0.37204 (19)	0.59235 (11)	0.0490 (5)	
C9	0.42638 (18)	0.3503 (2)	0.65930 (11)	0.0530 (5)	
C10	0.4029 (2)	0.4271 (3)	0.70306 (13)	0.0722 (8)	
H10	0.4077	0.4143	0.7473	0.090*	
C11	0.3730 (3)	0.5202 (3)	0.68277 (17)	0.0858 (10)	
H11	0.3569	0.5695	0.7133	0.090*	
C12	0.3661 (2)	0.5428 (2)	0.61802 (17)	0.0752 (8)	
H12	0.3463	0.6069	0.6045	0.090*	
C13	0.3893 (2)	0.4683 (2)	0.57297 (13)	0.0613 (7)	
H13	0.3860	0.4834	0.5289	0.090*	
C14	0.54177 (18)	0.2032 (2)	0.65208 (11)	0.0534 (6)	
C15	0.62253 (18)	0.2577 (2)	0.62606 (12)	0.0556 (6)	
H15	0.6210	0.3280	0.6266	0.090*	
C16	0.7052 (2)	0.2078 (3)	0.59926 (15)	0.0754 (9)	
H16	0.7585	0.2449	0.5810	0.090*	
C17	0.7097 (3)	0.1044 (4)	0.59921 (17)	0.0973 (12)	
H17	0.7665	0.0717	0.5818	0.090*	
C18	0.6305 (4)	0.0490 (3)	0.62469 (18)	0.0957 (12)	
H18	0.6334	−0.0212	0.6241	0.090*	
C19	0.5460 (3)	0.0972 (2)	0.65142 (15)	0.0754 (8)	
H19	0.4924	0.0595	0.6688	0.090*	
C20	0.4310 (2)	0.2244 (3)	0.74653 (14)	0.0802 (9)	
H20A	0.4540	0.1567	0.7548	0.090*	
H20B	0.3582	0.2283	0.7532	0.090*	
H20C	0.4650	0.2702	0.7756	0.090*	
C21	0.67820 (17)	0.32672 (17)	0.34710 (10)	0.0428 (5)	
H21	0.6876	0.2690	0.3220	0.090*	
C22	0.71833 (16)	0.42188 (18)	0.32147 (11)	0.0449 (5)	
C23	0.72020 (16)	0.43670 (19)	0.25358 (11)	0.0480 (5)	
C24	0.77257 (19)	0.5197 (2)	0.22871 (13)	0.0561 (6)	
H24	0.7750	0.5299	0.1841	0.090*	
C25	0.82048 (19)	0.5866 (2)	0.26927 (15)	0.0632 (7)	
H25	0.8567	0.6405	0.2516	0.090*	
C26	0.81626 (18)	0.5757 (2)	0.33613 (14)	0.0595 (6)	
H26	0.8470	0.6231	0.3632	0.090*	
C27	0.76565 (18)	0.4933 (2)	0.36163 (12)	0.0526 (6)	
H27	0.7629	0.4851	0.4064	0.090*	
C28	0.56550 (18)	0.34379 (18)	0.22359 (10)	0.0486 (5)	
C29	0.5025 (2)	0.4097 (2)	0.25693 (13)	0.0566 (6)	
H29	0.5288	0.4705	0.2724	0.090*	
C30	0.3998 (2)	0.3856 (3)	0.26745 (15)	0.0700 (8)	
H30	0.3583	0.4297	0.2909	0.090*	
C31	0.3597 (2)	0.2980 (3)	0.24368 (17)	0.0771 (9)	
H31	0.2909	0.2826	0.2502	0.090*	
C32	0.4222 (3)	0.2325 (3)	0.20982 (18)	0.0840 (9)	
H32	0.3950	0.1726	0.1936	0.090*	
C33	0.5235 (2)	0.2540 (2)	0.19977 (16)	0.0716 (8)	
H33	0.5646	0.2088	0.1770	0.090*	
C34	0.7164 (2)	0.3395 (2)	0.15165 (12)	0.0630 (7)	
H34A	0.6741	0.2906	0.1300	0.090*	
H34B	0.7835	0.3115	0.1586	0.090*	
H34C	0.7219	0.3990	0.1253	0.090*	

### Atomic displacement parameters (Å^2^)

**Table:**

	*U*^11^	*U*^22^	*U*^33^	*U*^12^	*U*^13^	*U*^23^
Fe1	0.04618 (16)	0.04382 (15)	0.03736 (16)	−0.00461 (14)	0.00017 (14)	−0.00167 (13)
Cl1	0.0632 (3)	0.0743 (4)	0.0513 (3)	−0.0149 (3)	−0.0097 (3)	−0.0089 (3)
Cl2	0.0795 (4)	0.0616 (4)	0.0597 (4)	0.0074 (3)	−0.0054 (3)	0.0132 (3)
N1	0.0368 (8)	0.0509 (9)	0.0329 (9)	−0.0012 (8)	−0.0005 (7)	−0.0017 (7)
N2	0.0381 (9)	0.0468 (9)	0.0332 (9)	−0.0033 (8)	0.0024 (7)	0.0020 (8)
N3	0.0528 (12)	0.0846 (12)	0.0379 (10)	−0.0104 (11)	0.0079 (9)	0.0083 (10)
N4	0.0578 (11)	0.0741 (13)	0.0378 (10)	−0.0103 (11)	0.0137 (9)	−0.0027 (10)
C1	0.0380 (10)	0.0486 (11)	0.0316 (11)	−0.0053 (9)	−0.0035 (8)	−0.0027 (8)
C2	0.0372 (10)	0.0438 (11)	0.0311 (11)	−0.0044 (9)	−0.0003 (8)	0.0032 (8)
C3	0.0572 (14)	0.0499 (13)	0.0448 (13)	0.0004 (11)	0.0026 (11)	−0.0015 (10)
C4	0.0765 (18)	0.0461 (12)	0.0565 (14)	−0.0036 (13)	0.0015 (13)	−0.0069 (11)
C5	0.0762 (17)	0.0527 (13)	0.0625 (17)	−0.0221 (13)	0.0004 (13)	−0.0053 (12)
C6	0.0529 (13)	0.0542 (12)	0.0482 (13)	−0.0153 (11)	0.0038 (11)	0.0003 (10)
C7	0.0361 (9)	0.0568 (11)	0.0361 (11)	−0.0058 (10)	0.0008 (10)	0.0039 (9)
C8	0.0379 (11)	0.0658 (14)	0.0432 (12)	0.0008 (11)	0.0059 (10)	−0.0051 (11)
C9	0.0391 (11)	0.0815 (13)	0.0385 (12)	−0.0058 (12)	0.0052 (10)	−0.0067 (11)
C10	0.0643 (16)	0.109 (2)	0.0431 (14)	−0.0046 (18)	0.0051 (11)	−0.0205 (16)
C11	0.077 (2)	0.106 (3)	0.074 (2)	0.020 (2)	0.0004 (16)	−0.043 (2)
C12	0.0716 (18)	0.0737 (19)	0.080 (2)	0.0170 (15)	−0.0006 (15)	−0.0148 (16)
C13	0.0557 (14)	0.0734 (16)	0.0549 (15)	0.0050 (13)	0.0040 (12)	−0.0078 (13)
C14	0.0522 (13)	0.0743 (15)	0.0335 (12)	−0.0071 (13)	−0.0066 (10)	0.0073 (11)
C15	0.0426 (12)	0.0819 (17)	0.0422 (13)	−0.0105 (12)	−0.0049 (10)	0.0031 (12)
C16	0.0499 (15)	0.122 (3)	0.0544 (17)	0.0069 (18)	−0.0055 (13)	0.0040 (18)
C17	0.093 (2)	0.143 (4)	0.0558 (19)	0.050 (3)	−0.0125 (18)	0.003 (2)
C18	0.132 (3)	0.089 (2)	0.067 (2)	0.035 (2)	−0.018 (2)	0.0068 (18)
C19	0.100 (2)	0.0679 (17)	0.0582 (17)	−0.0066 (17)	−0.0133 (16)	0.0156 (13)
C20	0.0707 (17)	0.124 (3)	0.0462 (15)	−0.0200 (19)	0.0112 (14)	0.0218 (16)
C21	0.0436 (11)	0.0499 (11)	0.0348 (11)	−0.0003 (10)	0.0023 (9)	0.0023 (9)
C22	0.0402 (11)	0.0511 (12)	0.0432 (12)	−0.0032 (11)	0.0079 (9)	0.0032 (11)
C23	0.0410 (10)	0.0596 (13)	0.0434 (12)	0.0028 (11)	0.0097 (9)	0.0013 (11)
C24	0.0546 (13)	0.0602 (15)	0.0535 (15)	−0.0043 (12)	0.0110 (11)	0.0140 (12)
C25	0.0465 (12)	0.0663 (16)	0.0767 (19)	−0.0040 (13)	0.0122 (12)	0.0160 (14)
C26	0.0462 (12)	0.0576 (13)	0.0748 (18)	−0.0101 (13)	0.0001 (12)	−0.0028 (14)
C27	0.0442 (12)	0.0638 (14)	0.0499 (14)	−0.0023 (11)	0.0044 (10)	0.0017 (12)
C28	0.0535 (12)	0.0562 (12)	0.0360 (11)	−0.0035 (12)	0.0073 (10)	−0.0001 (9)
C29	0.0557 (13)	0.0644 (15)	0.0495 (14)	−0.0069 (12)	0.0031 (11)	−0.0041 (12)
C30	0.0477 (13)	0.094 (2)	0.0688 (19)	−0.0002 (15)	0.0054 (13)	−0.0116 (16)
C31	0.0597 (16)	0.092 (2)	0.079 (2)	−0.0274 (16)	0.0053 (15)	−0.0091 (18)
C32	0.075 (2)	0.084 (2)	0.093 (2)	−0.0273 (18)	0.0032 (19)	−0.0236 (18)
C33	0.0713 (18)	0.0674 (16)	0.076 (2)	−0.0087 (15)	0.0074 (15)	−0.0168 (15)
C34	0.0601 (14)	0.0901 (19)	0.0388 (13)	0.0146 (15)	0.0097 (11)	0.0029 (13)

### Geometric parameters (Å, °)

**Table:**

Fe1—N2	2.1157 (18)	C13—H13	0.9300
Fe1—N1	2.1227 (18)	C14—C15	1.384 (4)
Fe1—Cl2	2.2408 (8)	C14—C19	1.403 (4)
Fe1—Cl1	2.2468 (7)	C15—C16	1.379 (4)
N1—C7	1.273 (3)	C15—H15	0.9300
N1—C1	1.487 (3)	C16—C17	1.369 (6)
N2—C21	1.272 (3)	C16—H16	0.9300
N2—C2	1.496 (3)	C17—C18	1.370 (6)
N3—C9	1.417 (3)	C17—H17	0.9300
N3—C14	1.422 (3)	C18—C19	1.387 (5)
N3—C20	1.459 (3)	C18—H18	0.9300
N4—C23	1.406 (3)	C19—H19	0.9300
N4—C28	1.419 (3)	C20—H20A	0.9600
N4—C34	1.450 (3)	C20—H20B	0.9600
C1—C6	1.523 (3)	C20—H20C	0.9600
C1—C2	1.539 (3)	C21—C22	1.462 (3)
C1—H1	0.9800	C21—H21	0.9300
C2—C3	1.520 (3)	C22—C27	1.400 (4)
C2—H2	0.9800	C22—C23	1.413 (3)
C3—C4	1.523 (4)	C23—C24	1.391 (3)
C3—H3A	0.9700	C24—C25	1.368 (4)
C3—H3B	0.9700	C24—H24	0.9300
C4—C5	1.505 (4)	C25—C26	1.386 (4)
C4—H4A	0.9700	C25—H25	0.9300
C4—H4B	0.9700	C26—C27	1.378 (4)
C5—C6	1.521 (4)	C26—H26	0.9300
C5—H5A	0.9700	C27—H27	0.9300
C5—H5B	0.9700	C28—C29	1.381 (3)
C6—H6A	0.9700	C28—C33	1.396 (4)
C6—H6B	0.9700	C29—C30	1.394 (4)
C7—C8	1.467 (3)	C29—H29	0.9300
C7—H7	0.9300	C30—C31	1.362 (5)
C8—C13	1.384 (4)	C30—H30	0.9300
C8—C9	1.414 (3)	C31—C32	1.379 (5)
C9—C10	1.393 (4)	C31—H31	0.9300
C10—C11	1.358 (5)	C32—C33	1.368 (4)
C10—H10	0.9300	C32—H32	0.9300
C11—C12	1.370 (5)	C33—H33	0.9300
C11—H11	0.9300	C34—H34A	0.9600
C12—C13	1.386 (4)	C34—H34B	0.9600
C12—H12	0.9300	C34—H34C	0.9600
			
N2—Fe1—N1	80.14 (7)	C8—C13—C12	121.2 (3)
N2—Fe1—Cl2	114.98 (5)	C8—C13—H13	119.4
N1—Fe1—Cl2	110.60 (5)	C12—C13—H13	119.4
N2—Fe1—Cl1	110.31 (5)	C15—C14—C19	119.2 (3)
N1—Fe1—Cl1	113.40 (5)	C15—C14—N3	121.7 (3)
Cl2—Fe1—Cl1	120.51 (3)	C19—C14—N3	119.2 (3)
C7—N1—C1	120.27 (18)	C16—C15—C14	120.0 (3)
C7—N1—Fe1	127.20 (15)	C16—C15—H15	120.0
C1—N1—Fe1	111.65 (12)	C14—C15—H15	120.0
C21—N2—C2	119.19 (18)	C17—C16—C15	120.8 (3)
C21—N2—Fe1	128.50 (15)	C17—C16—H16	119.6
C2—N2—Fe1	111.55 (12)	C15—C16—H16	119.6
C9—N3—C14	120.5 (2)	C16—C17—C18	120.1 (3)
C9—N3—C20	116.9 (2)	C16—C17—H17	119.9
C14—N3—C20	115.8 (2)	C18—C17—H17	120.0
C23—N4—C28	119.64 (18)	C17—C18—C19	120.3 (4)
C23—N4—C34	119.2 (2)	C17—C18—H18	119.8
C28—N4—C34	118.4 (2)	C19—C18—H18	119.8
N1—C1—C6	115.56 (17)	C18—C19—C14	119.6 (3)
N1—C1—C2	107.62 (16)	C18—C19—H19	120.2
C6—C1—C2	110.85 (18)	C14—C19—H19	120.2
N1—C1—H1	107.5	N3—C20—H20A	109.5
C6—C1—H1	107.5	N3—C20—H20B	109.5
C2—C1—H1	107.5	H20A—C20—H20B	109.5
N2—C2—C3	116.49 (17)	N3—C20—H20C	109.5
N2—C2—C1	107.68 (16)	H20A—C20—H20C	109.5
C3—C2—C1	111.54 (17)	H20B—C20—H20C	109.5
N2—C2—H2	106.9	N2—C21—C22	125.3 (2)
C3—C2—H2	106.9	N2—C21—H21	117.4
C1—C2—H2	106.9	C22—C21—H21	117.4
C2—C3—C4	110.92 (19)	C27—C22—C23	118.9 (2)
C2—C3—H3A	109.5	C27—C22—C21	121.7 (2)
C4—C3—H3A	109.5	C23—C22—C21	118.9 (2)
C2—C3—H3B	109.5	C24—C23—N4	122.5 (2)
C4—C3—H3B	109.5	C24—C23—C22	118.9 (2)
H3A—C3—H3B	108.0	N4—C23—C22	118.6 (2)
C5—C4—C3	110.7 (2)	C25—C24—C23	120.6 (2)
C5—C4—H4A	109.5	C25—C24—H24	119.7
C3—C4—H4A	109.5	C23—C24—H24	119.7
C5—C4—H4B	109.5	C24—C25—C26	121.5 (2)
C3—C4—H4B	109.5	C24—C25—H25	119.3
H4A—C4—H4B	108.1	C26—C25—H25	119.3
C4—C5—C6	110.7 (2)	C27—C26—C25	118.7 (2)
C4—C5—H5A	109.5	C27—C26—H26	120.7
C6—C5—H5A	109.5	C25—C26—H26	120.7
C4—C5—H5B	109.5	C26—C27—C22	121.3 (2)
C6—C5—H5B	109.5	C26—C27—H27	119.3
H5A—C5—H5B	108.1	C22—C27—H27	119.3
C5—C6—C1	111.74 (19)	C29—C28—C33	118.6 (2)
C5—C6—H6A	109.3	C29—C28—N4	121.3 (2)
C1—C6—H6A	109.3	C33—C28—N4	120.1 (2)
C5—C6—H6B	109.3	C28—C29—C30	120.3 (3)
C1—C6—H6B	109.3	C28—C29—H29	119.9
H6A—C6—H6B	107.9	C30—C29—H29	119.9
N1—C7—C8	124.2 (2)	C31—C30—C29	120.5 (3)
N1—C7—H7	117.9	C31—C30—H30	119.7
C8—C7—H7	117.9	C29—C30—H30	119.7
C13—C8—C9	119.4 (2)	C30—C31—C32	119.3 (3)
C13—C8—C7	120.5 (2)	C30—C31—H31	120.3
C9—C8—C7	120.0 (2)	C32—C31—H31	120.3
C10—C9—C8	117.7 (3)	C33—C32—C31	121.1 (3)
C10—C9—N3	122.4 (2)	C33—C32—H32	119.5
C8—C9—N3	119.9 (2)	C31—C32—H32	119.5
C11—C10—C9	121.7 (3)	C32—C33—C28	120.2 (3)
C11—C10—H10	119.1	C32—C33—H33	119.9
C9—C10—H10	119.1	C28—C33—H33	119.9
C10—C11—C12	121.1 (3)	N4—C34—H34A	109.5
C10—C11—H11	119.5	N4—C34—H34B	109.5
C12—C11—H11	119.5	H34A—C34—H34B	109.5
C11—C12—C13	118.9 (3)	N4—C34—H34C	109.5
C11—C12—H12	120.6	H34A—C34—H34C	109.5
C13—C12—H12	120.6	H34B—C34—H34C	109.5
